# Depression, anxiety and insomnia in Chinese older adults and their family caregivers during the COVID-19 pandemic: an actor-partner interdependence model approach

**DOI:** 10.3389/fpubh.2023.1163867

**Published:** 2023-06-27

**Authors:** Kai-Rong Ding, Wen-Qi Xu, Yong-Yi He, Jia-Hui Hu, Wen-Yan Tan, Jing Liao, Cai-Lan Hou, Fu-Jun Jia, Shi-Bin Wang

**Affiliations:** ^1^Guangdong Mental Health Center, Guangdong Provincial People's Hospital (Guangdong Academy of Medical Sciences), Southern Medical University, Guangzhou, China; ^2^Department of Psychology, School of Public Health, Southern Medical University, Guangzhou, China; ^3^Department of Medical Statistics, School of Public Health, Sun Yat-sen University, Guangzhou, China

**Keywords:** depression, anxiety, insomnia, older adults, actor-partner interdependence model

## Abstract

**Aims:**

This study aimed to explore the dyadic effects of depression and anxiety on insomnia symptoms in Chinese older adults and their caregivers living in a community setting.

**Methods:**

Data were collected from 1,507 pairs of older adults and their caregivers who were in the Guangdong Mental Health Survey in China. The 9-item Patient Health Questionnaire (PHQ-9), Generalized Anxiety Disorder module 7 (GAD-7), and Insomnia Severity Index (ISI) were used to measure depression, anxiety, and insomnia symptoms. Actor-Partner Interdependence Models (APIM) were used to determine whether anxiety or depression symptoms predicted individual or dyadic insomnia.

**Results:**

Older adults' and caregivers' depression and anxiety had significant positive correlations with their own and their caregivers' insomnia symptoms (all *P* < 0.001). Actor effects were found between depression and insomnia symptoms in both older adults and caregivers (B = 0.695, *P* < 0.001; B = 0.547, *P* < 0.001, respectively), with one significant partner effects (B = 0.080, *P* = 0.007). Actor effects were also found between anxiety and insomnia symptoms in both older adults and caregivers (B = 0.825, *P* < 0.001; B = 0.751, *P* < 0.001, respectively), with one significant partner effects (B = 0.097, *P* = 0.004). However, the caregivers' depression and anxiety were not associated with older adults' insomnia symptoms in the APIM analyses.

**Conclusions:**

Older adults and their caregivers had an interrelationship between psychological distress and insomnia. Consequently, healthcare providers might consider involving dyads when designing programs to reduce insomnia and improve psychological distress for family caregivers.

## 1. Introduction

China bears a heavy burden of disease due to its large population of older adults aged 65 or more, which accounts for 13.5% of the total population (1). China is already amongst the aging societies and the burden of diseases has subsequently risen as a result (2). Sleep disturbance, depressive and anxiety disorders are relatively prevalent psychiatric conditions in late life. A meta-analysis of older people in Europe and North America illustrated a lifetime prevalence of major depression of 16.5% (3). Similarly, a systematic review showed that the point prevalence of late-life mild depression varies from 0 to 18.6% (4). According to former findings, the current and lifetime prevalence rates of anxiety disorders among older adults were 14.2 and 30.0%, respectively (5). In addition, over half of older adults worldwide experienced trouble sleeping, and 20 to 40% reported insomnia (6). Mental health problems not only worsened older adults' quality of life and social function, but also increased caregiver burden.

Owing to the Chinese traditional Confucian contexts, China places a great importance on respecting and caring for older adults, as well as an old age of want. In China, older adults are generally living with spouse and/or children, which provides material, emotional, and other supports to older adults and promote interaction with family members (7). Family members are main providers of care for the Chinese older adults (8, 9). This brings the multiple burdens to family caregivers, including objective burdens (caregiving-related negative things and changes in physical health and behavior) and subjective burdens (emotional reactions such as anxiety, worry, frustration, and fatigue) (10). With the COVID-19 pandemic, quarantine was considered as a protective strategy and has a radical impact on older adults' lifestyle (11). Older adults were required to stay at home, it increases the caregiving load (12) and exacerbates generational family conflict with caregivers (13). Older adults had a higher risk of COVID-19 infection and mortality rate than other individuals, which can detriment their mental health (14, 15). Some studies showed that older adults with chronic disease and their family caregivers experienced more psychological distress, such as depression, anxiety and sleep problems (16–18). Many studies on mental health at work concentrated on the anxiety and depressive symptoms of caregivers (19). Previous research displayed that 46.8 and 29.8% of older adults' caregivers had anxiety and depressive symptoms during the COVID-19 outbreak (15). Another undesirable outcome of long-term care is insomnia. Financial strain, limited leisure time, social isolation, and somatic condition of cared-for people may contribute to psychological distress and insomnia of caregivers (20–23).

Multiple studies have examined the relationship between psychological distress and sleep problems in older adults and caregivers (24–27). But older adults-caregiver dyadic associations between psychiatric symptomatology and insomnia have not received adequate attention. Although anxiety, depression and sleep problems were often studied on an individual level, the effect of interpersonal factors has increasingly become the focus of researchers in recent years (28, 29). Analyses of the dyadic data indicated inter-person correlations between chronic disease patients' and their caregivers' emotions (e.g., anxiety, depression) (16–18). Similarly, the congruence in emotions and sleep has also been reported in cancer patients and their spouses (30).

However, very few studies focused on general aged population, and how the dyadic relationship of depression and anxiety affects insomnia among Chinese older adults and their family caregivers was even rarely mentioned in COVID-19 outbreak. This gap in the literature potentially restricts the development and application of dyad-based interventions. Therefore, this study aimed at examining whether there is a dyadic relationship between depression, anxiety and insomnia among Chinese older adults and their family caregivers during the COVID-19 outbreak.

We proposed three hypotheses as follows:

1. Hypothesis 0: Individuals' depression and anxiety were positively associated with their own insomnia (actor effects).
2. Hypothesis 1: Older adults' depression and anxiety were positively associated with their caregivers' insomnia (partner effects).
3. Hypothesis 2: Caregivers' depression and anxiety were positively associated with older adults' insomnia (partner effects).

## 2. Methods

### 2.1. Participants and procedure

Participants in this study were older adult-caregiver dyads drawn from the Guangdong Mental Health Survey in China, which was conducted from October 2021 to December 2021. The target sample was picked from community-dwelling residents adopting multistage stratified cluster sampling, which included all the 21 administrative regions of Guangdong province. In the first stage, probability proportional to size (PPS) sampling was applied to choose 3 to 5 districts or counties from each administrative region. In the second stage, based on the population size of each district or county, we chose 1 to 4 subdistricts or towns from each selected district or county. Subsequently, we chose 2 to 4 village councils or neighborhood committees from each subdistrict or town using PPS, and then 50 residents were picked from each neighborhood. Finally, one adult resident older than 18 years was randomly chosen from each family in the selected village council or neighborhood committee. The sample included 18,464 residents aged 18 years and above, of whom 4,018 were aged 65 or over and completed the survey. For the selected older adults, their family caregivers were invited to participate in this survey. For this study, the definition of family caregivers is family members who provides care (daily life) for older adults.

In this study, the inclusion criteria of older adults were: (1) community-living older adults aged 65 years or older, (2) fluent Chinese language speaking, (3) provided informed consent, and (4) the selected older adults have caregivers who agreed to participate in this study and provided an informed assent. Exclusion criteria were: (1) residing in a nursing home, (2) hospitalization during the study period.

A total of 1,507 dyads provided complete data out of the 4,018 older individuals who were invited to participate in this study. Thus, 1,507 dyads in total were included for dyadic analyses. The questionnaire had a 37.5% response to invitation rate.

Socio-demographics, health-related factors, and mental health were interviewed face-to-face by investigators who have gone through uniform training, using an electronic structured questionnaire at local health service centers. The Research protocol was approved by the Ethics Committee of the Guangdong Provincial People's Hospital, Guangdong Academy of Medical Sciences (Reference number: KY2020-268-01).

### 2.2. Measures

#### 2.2.1. Mental health characteristics

Older adults and caregivers rated their own depression symptoms in the past year using the 9-item Patient Health Questionnaire (PHQ-9). The PHQ-9, a self-reported 4-point Likert scale, contained nine items that measure depression in the past years. The total score ranges from 0 to 27, with higher scores representing worse depressive symptoms. A total score of 5 or more was considered as having depression symptoms (31). The Chinese version of PHQ-9 has already been used in many studies and proved to be reliable and valid (32, 33). We use Cronbach's alpha coefficient to assess internal consistency reliability of the questionnaires. In this study population, Cronbach's alpha coefficient of PHQ-9 was 0.940 for older adults and 0.924 for caregivers.

The Generalized Anxiety Disorder-7 item (GAD-7) (34) was used to measure older adults' and caregivers' anxiety symptoms in the past year. The GAD-7 contains seven items rated on a 4-point Likert scale, measuring anxiety in the past years. The total score ranges from 0 to 21, and each item rated a score of 0 to 3 (“not at all,” “several days,” “more than half the days,” “almost every day”). GAD-7 score of 5 or more was considered having anxiety symptoms. Scores are summed, with higher scores indicating more serious anxiety. It has been proved to have acceptable reliability and validity in the Chinese population (35). In the current study, the Cronbach's alpha coefficient of GAD-7 for older adults and their caregivers were 0.917 and 0.894, respectively.

Older adults and caregivers reported their insomnia symptoms in the past month using Insomnia Severity Index (ISI) (36). The ISI embodies seven items scored from o to 4, assessing the severity of insomnia. The total score ranges from 0 to 21, with higher scores indicating more severe insomnia and a score of 7 or more representing insomnia symptoms (37, 38). The scale has shown good psychometric properties in China (39). In this study, the Cronbach's alpha coefficient of ISI was 0.929 and 0.893 for older adults and caregivers, respectively.

#### 2.2.2. Socio-demographics and health-related factors

The socio-demographics data include age, gender, region (urban/rural), education status, monthly income, marital status, and occupation group. Education level was recoded into five categories: primary school or below, junior high school, senior high school, college, and graduate school or above. Monthly income was categorized into 4 classes: <3,500, 3,500–5,999, 6,000–8,999, >9,000 ¥. Occupation group was divided into four categories: government officer/teacher/healthcare provider, factory/business/agriculture/service industry employee, retired, and others (40, 41). Marital status was dichotomized: married/cohabitating, and single/widowed/divorced/separated. Smoking status was divided into two groups: not smoking (including past smoking) and current smoking. Current smoking was defined as smoking at least one cigarette per day and continuing to smoke in the past 6 months. Alcohol consumption was defined as drinking at least once a week. The definition of tea-drinking habits was those of participants drinking tea at least four times a week. The exercise habits in the past year were grouped into five levels: hardly exercise, occasionally exercise (1–3 times/month), sometimes exercise (1–2 times/week), often exercise (more than 3 times/week), and almost every day. Chronic diseases were self-report of doctor-diagnosis and were listed based on International Classification of Disease, 10th revision (ICD-10), which contained hypertension, diabetes, cardio vascular disease, cerebrovascular disease, chronic obstructive pulmonary disease (COPD), hyperlipidemia, arthritis, discogenic diseases, chronic gastroenteritis/peptic ulcer, gallstones/chronic cholecystitis, urolithiasis, chronic hepatitis/hepatocirrhosis, cataract/glaucoma, gout, nervous system disease, cancer and anemia. Participants were asked “In the past year, have you ever been diagnosed by a physician that you have any of chronic disease in the list?” The number of chronic diseases was calculated and divided into three groups: 0 (no chronic disease), 1 (one chronic disease), and ≥2 (the co-occurrence of two or more chronic diseases) (42).

### 2.3. Statistical analyses

The paired sample *t*-tests, McNemar tests and McNemar-Bowker tests were used to compare characteristics between older adults and caregivers. Spearman bivariate correlations were conducted to examine older adult-caregiver associations between all primary variables. An actor-partner interdependency model (APIM) was used to examine the actor and partner effects on insomnia. Actor effects refer to whether a person's PHQ-9 score is related to their own ISI score. Partner effects mean the association between an individual's PHQ-9 score and another individual's ISI score. We specified older adults' and caregivers' depressive symptoms and anxiety symptoms as predictors, respectively, and their insomnia symptoms as outcome variables in the APIM analyses. In the final model, older adults' and caregivers' age, gender, and region were added as covariates. Restricted maximum likelihood estimation methods were applied. Model fit was evaluated by using the chi-square statistic, root mean square error of approximation (RMSEA), comparative fit index (CFI), standardized root mean residual (SRMR), and goodness of fit index (GFI). APIM models were performed in AMOS 24.0 and the rest of data analyses were performed in SPSS 25.0. All statistical tests were two-tailed with the level of significance set as 0.05.

## 3. Results

### 3.1. Characteristics description

A total of 1,507 older adults and their corresponding primary caregivers participated in this study (3,014 individuals). Table 1 reports the distribution of older adults' and caregivers' characteristics.

**Table 1:** Sociodemographic, health related factors and mental health characteristics of older adults and their caregivers (*N* = 1,507 dyads).

**Variables**	**Older adults**	**Caregivers**	**McNemar**	***P***
	***n*** **(%)**	***n*** **(%)**		
**Gender**			**8.455**	**0.004**
Female	787 (52.2)	869 (57.7)		
Male	720 (47.8)	638 (42.3)		
**Region**			**1.235**	**0.267**
Rural	732 (48.6)	721 (47.8)		
Urban	775 (51.4)	786 (52.2)		
**Education**			**786.387**	**<0.001**
Primary school or lower	910 (60.4)	219 (14.5)		
Junior high school	338 (22.4)	452 (30.0)		
Senior high school	201 (13.3)	343 (22.8)		
College or higher	58 (3.8)	493 (32.7)		
**Income (RMB)**			**146.445**	**<0.001**
<3,500	927 (61.5)	693 (46.0)		
3,500–5,999	367 (24.4)	478 (31.7)		
6,000–9,000	100 (6.6)	162 (10.7)		
>9,000	113 (7.5)	174 (11.5)		
**Occupation**			**578.237**	**<0.001**
Government officer/teacher/healthcare provider	20 (1.3)	250 (16.6)		
Factory/business/agriculture/service industry employee	694 (46.1)	659 (43.7)		
Retired	579 (38.4)	115 (7.6)		
Other	214 (14.2)	483 (32.1)		
**Marital status**			**95.608**	**<0.001**
Married/cohabitation	1,114 (73.9)	1,329 (88.2)		
Single/widowed/divorced/separated	393 (26.1)	178 (11.8)		
Current smoking			0.110	0.740
Yes	280 (18.6)	288 (19.1)		
No	1,227 (81.4)	1,219 (81.9)		
**Current alcohol drinker**			**0.938**	**0.333**
Yes	114 (7.6)	129 (8.6)		
No	1,393 (92.4)	1,378 (91.4)		
**Tea-drinking habits**			**6.840**	**0.009**
Yes	709 (47.0)	771 (51.2)		
No	898 (53.0)	736 (48.8)		
**Exercise frequency**			**251.626**	**<0.001**
Hardly ever	362 (24.0)	299 (19.8)		
1–3 times/month	105 (7.0)	301 (20.0)		
1–2 times/week	126 (8.4)	272 (18.0)		
3–5 times/week	138 (9.2)	161(10.7)		
Almost everyday	776 (51.5)	474 (31.5)		
**Number of chronic diseases**			**458.264**	**<0.001**
0	504 (33.4)	1,089 (72.3)		
1	529 (34.4)	279 (18.5)		
≥2	484 (32.1)	139 (9.2)		
	**Mean (SD)**	**Mean (SD)**	**t** ^a^	***P***
Age (years)	72.6 (6.3)	45.7 (12.6)	78.059	**<0.001**
Depression	0.99 (2.82)	1.15 (2.89)	−1.663	0.097
Anxiety	1.03 (2.43)	1.16 (2.45)	−1.654	0.098
Insomnia	3.37 (4.24)	2.85 (3.62)	4.043	**<0.001**

Findings significant at the P <0.05 level are shown in bold. ^*a*^Paired sample t-test.

### 3.2. Bivariate correlations

All bivariate correlations are presented in Table 2. For the older adults, their insomnia was significantly positively correlated to their own depression (*r* = 0.397, *P* < 0.001) and anxiety (*r* = 0.450, *P* < 0.001), and was also correlated to their caregivers' depression (*r* = 0.135, *P* < 0.001) and anxiety (*r* = 0.167, *P* < 0.001). In addition, the older adults' depression, anxiety and insomnia were associated with the caregiver's depression (*r* = 0.238, *P* < 0.001), anxiety (*r* = 0.271, *P* < 0.001), and insomnia (*r* = 0.247, *P* < 0.001), respectively. The levels of older adult-caregiver correlation among depression, anxiety, and insomnia were lower than those of intra-subject correlation (*r* = 0.135 to 0.271 vs. *r* = 0.397 to 0.587, respectively).

**Table 2:** Bivariate correlation matrices of depression, anxiety and insomnia for older adults and caregivers (r).

**Variables**	**1**	**2**	**3**	**4**	**5**	**6**
1. Older adults Depression	1.00					
2. Older adults Anxiety	0.541^***^	1.00				
3. Older adults Insomnia	0.397^***^	0.450^***^	1.00			
4. Caregivers Depression	0.238^***^	0.190^***^	0.135^***^	1.00		
5. Caregivers Anxiety	0.245^***^	0.271^***^	0.167^***^	0.587^***^	1.00	
6. Caregivers Insomnia	0.192^***^	0.199^***^	0.247^***^	0.411^***^	0.497^***^	1.00

^***^Significant at the 0.001 level (two-tailed).

### 3.3. Actor-partner interdependence models

Two hypotheses were examined and four APIMs were established (Table 3). The collective covariates associated with insomnia for the older adults and caregivers contained age, gender and region, which were ultimately considered into our models as control variables to help explain interdependence among older adults and caregivers' insomnia (*P* < 0.05). Screening results of covariates are shown in Supplementary material (Table S1).

**Table 3:** APIM results between older adults and caregivers for depression, anxiety and insomnia.

**Model**	**Paths**	**Estimate (B)**	**SE**	**95%CI**	***P***	**β**
**Model 1** ^**a**^	**Actor effect**				
	Older adults depression → older adults insomnia	0.695	0.035	0.558,0.817	**<0.001**	0.460
	Caregivers depression → caregivers insomnia	0.547	0.029	0.460,0.637	**<0.001**	0.446
	**Partner effect**				
	Older adults depression → caregivers insomnia	0.080	0.030	0.023,0.154	**0.005**	0.062
	Caregivers depression → older adults insomnia	0.040	0.034	−0.020,0.111	0.236	0.025
**Model 2** ^**b**^	**Actor effect**				
	Older adults depression → older adults insomnia	0.674	0.035	0.537,0.796	**<0.001**	0.450
	Caregivers depression → caregivers insomnia	0.557	0.029	0.470,0.648	**<0.001**	0.443
	**Partner effect**				
	Older adults depression → caregivers insomnia	0.083	0.030	0.025,0.156	**0.005**	0.064
	Caregivers depression → older adults insomnia	0.035	0.034	−0.024,0.106	0.293	0.024
**Model 3** ^**c**^	**Actor effect**				
	Older adults anxiety → older adults insomnia	0.825	0.041	0.676,0.963	**<0.001**	0.472
	Caregivers anxiety → caregivers insomnia	0.751	0.033	0.635,0.869	**<0.001**	0.508
	**Partner effect**				
	Older adults anxiety → caregivers insomnia	0.097	0.034	0.021,0.178	**0.004**	0.065
	Caregivers anxiety → older adults insomnia	0.028	0.040	−0.053,0.124	0.481	0.016
**Model 4** ^**d**^	**Actor effect**				
	Older adults anxiety → older adults insomnia	0.802	0.041	0.655,0.942	**<0.001**	0.462
	Caregivers anxiety → caregivers insomnia	0.749	0.033	0.639,0.865	**<0.001**	0.508
	**Partner effect**				
	Older adults anxiety → caregivers insomnia	0.093	0.033	0.018,0.173	**0.005**	0.063
	Caregivers anxiety → older adults insomnia	0.031	0.040	−0.050,0.126	0.477	0.018

APIM, Actor-Partner Interdependence Models; B, unstandardized coefficients; β, standardized coefficients; SE, standard error; 95% CI, 95% Bias Corrected Confidence Interval. Findings significant at the P <0.05 level are shown in bold. ^*a*^Model 1 is a crude model to examine the relationship between depression and insomnia without covariates. ^*b*^Model 2 is a covariates-adjusted model based on model 1 after controlling age, gender and region. ^*c*^Model 3 is a crude model to examine the relationship between anxiety and insomnia without covariates. ^*d*^Model 4 is a covariates-adjusted model based on model 3 after controlling age, gender and region.

**Figure 1:**
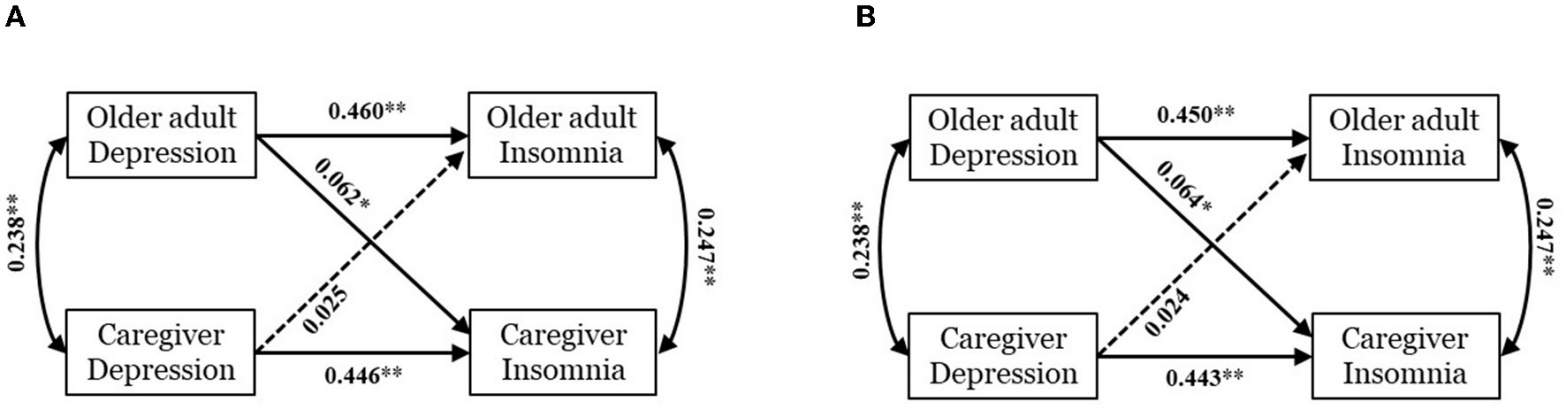
Actor–partner interdependence model of depression and insomnia within older adults-caregivers dyads. Model **(A)** is a crude model without covariates. Model **(B)** is an adjusted model controlling age, gender and region. Standardized coefficients (β) are presented on straight lines with single arrow and correlation coefficients are reported on curves with double-headed arrows. A solid line and dotted line represent significant path and non-significant one respectively. *p < 0.01; **p < 0.001.

Interdependence among older adults-caregivers dyads' depression and insomnia were explored without adjusting for covariates. Firstly, the overall Chi-square testing of distinguishability was significant ($\chi_{\left(2\right)}^{2}$ = 10.377, *P* = 0.006, CFI = 0.989, GFI = 0.997, RMSEA = 0.053, SRMR = 0.019), indicating that actor and partner effects were statistically distinguished between older adults and their caregivers. Accordingly, dyadic interdependence was further analyzed and illustrated in Figure 1A.

Older adults' and caregivers' more serious depression was related to their own more serious insomnia (B_Olderadults_ = 0.695, 95% CI: 0.558~0.817, *P* < 0.001; B_Caregivers_ = 0.547, 95% CI: 0.460~0.637, *P* < 0.001) and thus significant actor effects were found. In the partner effect, older adults' depression was associated with caregivers' insomnia (B = 0.080, 95% CI: 0.023~0.154, *P* = 0.005), whereas caregivers' depression was not associated with older adults' insomnia (B = 0.040, 95% CI:−0.020~0.100, *P* = 0.236).

After adjusting age, gender and region, the APIM performing impact of depression on insomnia was reconstructed as shown in Figure 1B. As with initial crude model (model 1), the actor and partner effects of adjusted model (model 2) were also distinguishable. Two actor effects and one partner effect have been identified: older adults' depression positively impacted their own and caregivers' insomnia (B_Olderadults_ = 0.674, 95% CI: 0.537~0.796, *P* < 0.001; B_Caregivers_ = 0.557, 95% CI: 0.470~0.648, *P* < 0.001), while caregivers' depression only had a positive effect on their own insomnia (B = 0.083, 95% CI: 0.025~0.156, *P* = 0.005), but not on older adults' insomnia (B = 0.035, 95% CI: −0.024~0.106, *P* = 0.293). More details of APIM results are summarized in Table 3.

**Figure 2:**
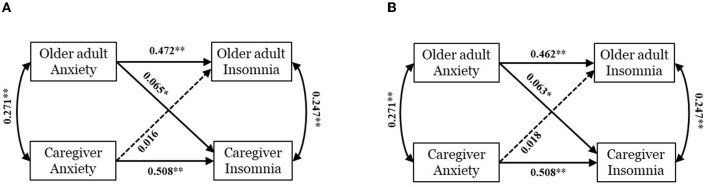
Actor–partner interdependence model of anxiety and insomnia within older adults-caregivers dyads. Model **(A)** is a crude model without covariates. Model **(B)** is an adjusted model controlling age, gender and region. Standardized coefficients (β) are presented on straight lines with single arrow and correlation coefficients are reported on curves with double-headed arrows. A solid line and dotted line represent significant path and non-significant one respectively. *p < 0.01; **p < 0.001.

We analyzed dyadic interdependence between older adults and caregivers about impact of anxiety on insomnia. The global model also displayed adequate fit ($\chi_{\left(2\right)}^{2}$ = 2.653, *P* = 0.265, CFI = 0.999, GFI = 0.999, RMSEA = 0.015, SRMR = 0.010). Greater older adults' and caregivers' anxiety was significantly correlated to their own greater insomnia (B_Olderadults_ = 0.825, 95% CI: 0.676~0.963, *P* < 0.001; B_Caregivers_ = 0.751, 95% CI: 0.635~0.869, *P* < 0.001). Furthermore, partner effect from older adults' anxiety to caregivers' insomnia was observed (B = 0.097, 95% CI: 0.021~0.178, *P* = 0.004), but corresponding effect from caregivers' anxiety to older adults' insomnia was not found (B = 0.028, 95% CI:−0.053~0.124, *P* = 0.481). Figure 2A and Table 3 show the coefficients and effects of each path.

Considering covariates, the adjusted model is depicted in Figure 2B and Table 3. We found two significant actor effects: older adults' and caregivers' anxiety worsen their own insomnia (B_Olderadults_ = 0.802, 95% CI: 0.655~0.942, *P* < 0.001; B_Caregivers_ = 0.749, 95% CI: 0.639~0.865, *P* < 0.001), and one partner effect of older adults' anxiety aggravated caregivers' insomnia (B = 0.093, 95% CI: 0.018~0.173, *P* = 0.005). No partner effect for caregivers' anxiety on older adults' insomnia was identified (B = 0.031, 95% CI: −0.050~0.126, *P* = 0.477).

## 4. Discussion

This study added to the evidence on the partner effects of depression and anxiety on insomnia in community-living older adults and their family caregivers in China. We found significant older adults-caregiver associations in depression, anxiety, and insomnia. Specifically, older adults' and caregivers' depression and anxiety had an influence on their own insomnia (actor effects); older adults' depression and anxiety had an influence on caregivers' insomnia (partner effects). Our findings indicate that it is feasible to embed emotional interventions for older adults within family care support services.

We found significant correlations in depression, anxiety and insomnia between older adults and caregivers, representing emotional contagion occurred in the family's environment (43), which is consistent with previous studies on patients with cancer (44). The psychological distress of patients and family caregivers are closely linked (45). These results could be explained by emotional contagion theory, in which emotional state can be transmitted from one person to another through nonverbal communication information during social interactions (46). Secondly, according to the interdependence theory, older adults and family caregivers are in the same social system, and they can influence each other (47). Stay-at-home orders during the COVID-19 outbreak increased family conflict and financial strain (48). Such stress may affect caregivers' sleep. Future study should pay attention to the nature of the interactions between the caregivers and the older adults in family environment other than material supports to older adults.

Consistent with earlier studies (30, 49), we found a significant actor effect of older adults' depression or anxiety on their own insomnia, which fully supports our hypothesis 0. Depression or anxiety in older adults can influence their sleep quality, and this also appears to be the case for caregivers. Present day research considers insomnia as a transdiagnostic symptom for many mental disorders, and it has been found that the relationship between depression and insomnia is bidirectional: people with depression have changes in sleep, and insomnia increases the risk of depression (50). Similar to depression, the APMI showed that more anxiety symptoms were associated with severe insomnia. Previous epidemiological research has shown that people with anxiety have a high prevalence of sleep problems (51). Anxiety is particularly characterized by a state of mental hyperarousal, which can display as a lower wake threshold, increasing the likelihood of sleep disturbance in reaction to external stimuli. Insomnia can be caused by false beliefs, bad sleep habits, somatic hyperarousal, and mental hyperarousal (52). Relaxation techniques used in Cognitive Behavioral Therapy for Insomnia (CBT-I) may help improve anxiety status by reducing hyperarousal (53). The successful use of relaxation techniques in CBT-I demonstrated that insomnia is caused by anxiety in certain extent.

The partial partner effect of depression or anxiety on insomnia was observed in this study: older adults' depression or anxiety had an effect on caregivers' insomnia, while caregivers' depression or anxiety had no effect on older adults' insomnia. Hypothesis 1 was supported and Hypothesis 2 was not supported. This is in line with the results of a previous study of patients and their carers (30). However, a former study in couples found that between-person effect was stronger than the within-person effect, and that anxiety symptoms predicted more aspects of sleep than depression (54). When an individual shared a bed with a partner, anxiety status might increase the risk of waking up, whereas depression, which is a psychomotor retardation, might not produce the same risk of waking up. In the current sample, caregivers are family member of older adults, including spouses, daughters, sons, kins or daughters-in-law. We cannot distinguish family caregivers' relationship to older adults. To remedy this defect of the study design, the covariate age was adjusted in APIM analyses due to the age of the spouse was greater than daughters, sons, kins or daughters-in-law. Though the main results did not change, these results should be viewed with some caution. Future research should focus on caregivers from specific gender group or different relationships, and use methods with better ecological validity and involve longitudinal and causal study designs. One possible reason why caregivers' depression or anxiety did not have a partner effect on older adults' insomnia is the difference in roles. Caregiving activities can disturb caregivers' daily lives, causing physical, emotional and financial stress, and finally consuming their energy (10). We guess, as care recipients, older adults are more likely to be influenced by the reality of caregivers' care activities than by the caregivers' emotions. Therefore, further research is needed to confirm our speculation.

Overall, this study added to the literature on psychological distress and insomnia within the older adults-caregiver dyads, and explored the between-person effects of emotion. Previous studies of psychological distress and insomnia among older adults and caregivers examined unidirectional effects instead of mutual effects in focus. However, care recipients and caregivers have a close relationship and may influence each other through emotional, cognitive and behavioral clues in social interactions, and may eventually affect the health of the caregivers (23, 55). There are several limitations in this study. First, we didn't distinguish the roles of informal family caregivers (spouse caregiver, son caregiver, or daughter caregiver). Second, Selection bias may exist in this study. The general demographics are significantly different between the two groups of older adults. For more information, see the Supplementary material (Table S2). The rest of 2,511 older adults did not report having caregiver or their caregiver declined to participate in this study. Third, the cross-sectional design did not allow us to produce causal relationships. Fourth, the results from the provincial data could not be generalized to the whole country.

## 5. Conclusion

Psychological distress in older adults may affect the sleep of their caregivers. Healthcare providers should provide mental health services based on family or group interaction. Our findings highlight the need for older adult-caregiver dyad interventions and strategies for managing older adults' emotions in the treatment of insomnia in family caregivers.

## Data availability statement

The raw data supporting the conclusions of this article will be made available by the authors, without undue reservation.

## Ethics statement

The studies involving human participants were reviewed and approved by the Ethics Committee of the Guangdong Provincial People's Hospital, Guangdong Academy of Medical Sciences (Reference number: KY2020-268-01). The patients/participants provided their written informed consent to participate in this study.

## Author contributions

K-RD: conceptualization, methodology, formal analysis, investigation, writing-original draft, and writing—review and editing. W-QX: methodology, writing-original draft, and writing—review and editing. Y-YH: formal analysis, investigation, and writing—review and editing. J-HH: writing-review and editing. W-YT: resources and supervision. JL: methodology. C-LH: conceptualization. F-JJ: conceptualization, resources, and supervision. S-BW: conceptualization, methodology, investigation, writing-original draft, and writing—review and editing. All authors contributed to the article and approved the submitted version.
