# Supplementary material for: Depression, anxiety and insomnia in Chinese older adults and their family caregivers during the COVID-19 pandemic: an actor-partner interdependence model approach
Source: Front Public Health. 2023 Jun 27;11:1163867. doi: 10.3389/fpubh.2023.1163867 (PMC10333500; doi:10.3389/fpubh.2023.1163867)
Supplement: Supplementary file 1 [file Table_1.DOCX]

Supplementary Material 1

| Table S1. Demographics of caregivers and older adults on Insomnia | | | | | | | | |
| --- | --- | --- | --- | --- | --- | --- | --- | --- |
| Variables | Caregiver Insomnia | | | | Older adults Insomnia | | | |
|  | Total | M (SD) | *t/F* | *P* | Total | M (SD) | *t/F* | *P* |
| Age (years) |  |  |  |  |  |  |  |  |
| 18-44 | 817 | 2.57 (3.18) | 5.608 | **0.004** | - | - | - | - |
| 45-64 | 523 | 3.17 (3.99) |  |  | - | - | - | - |
| ≥65 | 167 | 3.31 (4.40) |  |  | - | - | - | - |
| 65-70 | - | - | - | - | 696 | 3.11 (3.93) | -2.215 | **0.027** |
| >70 | - | - | - | - | 811 | 3.59 (4.48) |  |  |
| Gender |  |  |  |  |  |  |  |  |
| Female | 869 | 3.05 (3.85) | 2.588 | **0.010** | 787 | 3.85 (4.59) | 4.614 | **<0.001** |
| Male | 638 | 2.58 (3.27) |  |  | 720 | 2.85 (3.76) |  |  |
| Region |  |  |  |  |  |  |  |  |
| Rural | 721 | 2.59 (3.50) | -2.728 | **0.006** | 732 | 3.07 (4.08) | -2.698 | **0.007** |
| Urban | 786 | 3.09 (3.72) |  |  | 775 | 3.66 (4.37) |  |  |
| Education |  |  |  |  |  |  |  |  |
| Primary school or lower | 219 | 3.28 (4.11) | 1.282 | 0.279 | 910 | 3.58 (4.42) | 1.876 | 0.132 |
| Junior high school | 452 | 2.75 (3.69) |  |  | 338 | 3.00 (4.10) |  |  |
| Senior high school | 343 | 2.73 (3.62) |  |  | 201 | 3.08 (3.64) |  |  |
| College or higher | 493 | 2.84 (3.32) |  |  | 58 | 3.28 (3.90) |  |  |
| Income (RMB) |  |  |  |  |  |  |  |  |
| <3500 | 693 | 3.07 (3.91) | 2.084 | 0.100 | 927 | 3.32 (4.29) | 0.530 | 0.662 |
| 3500-5999 | 478 | 2.55 (3.29) |  |  | 367 | 3.30 (4.27) |  |  |
| 6000-9000 | 162 | 2.69 (3.17) |  |  | 100 | 3.61 (3.82) |  |  |
| >9000 | 174 | 2.97 (3.68) |  |  | 113 | 3.78 (4.11) |  |  |
| Marital status |  |  |  |  |  |  |  |  |
| Married/cohabitation | 1329 | 2.82 (3.63) | -0.813 | 0.417 | 1114 | 3.08 (3.99) | -4.163 | **<0.001** |
| Single/widowed/divorced/separated | 178 | 3.06 (3.57) |  |  | 393 | 4.20 (4.80) |  |  |
| Occupation |  |  |  |  |  |  |  |  |
| Government officer /teacher/healthcare provider | 250 | 2.56 (3.20) | 0.920 | 0.431 | 20 | 3.60 (3.66) | 1.209 | 0.305 |
| Factory/business/agriculture/service industry employee | 659 | 2.84 (3.59) |  |  | 694 | 3.31 (4.27) |  |  |
| Retired | 115 | 3.17 (4.43) |  |  | 579 | 3.25 (3.96) |  |  |
| Other | 483 | 2.94 (3.67) |  |  | 214 | 3.87 (4.88) |  |  |
| Findings significant at the *P*<0.05 level are shown in bold. *t*-tests and one way ANOVA were used in the statistical analysis. | | | | | | | | |

| Table S2 Comparison of characteristics between older adults with caregiver and without caregiver (n=4018) | | | | |
| --- | --- | --- | --- | --- |
| Variables | With caregiver  n=1507 | Without caregiver  n=2511 | *χ^2^* | *P* |
|  | n (%) | n (%) |  |  |
| Gender |  |  | 7.947 | **0.005** |
| Female | 787 (52.2) | 1196 (47.6) |  |  |
| Male | 720 (47.8) | 1315 (52.4) |  |  |
| Region |  |  | 36.901 | **<0.001** |
| Rural | 732 (48.6) | 974 (38.8) |  |  |
| Urban | 755 (51.4) | 1537 (61.2) |  |  |
| Education |  |  | 41.578 | **<0.001** |
| Primary school or lower | 911 (60.5) | 1259 (50.1) |  |  |
| Junior high school | 338 (22.4) | 674 (26.8) |  |  |
| Senior high school | 200 (13.3) | 447 (17.8) |  |  |
| College or higher | 58 (3.8) | 131 (5.2) |  |  |
| Income (RMB) |  |  | 8.716 | **0.033** |
| <3500 | 928 (61.6) | 1484 (59.1) |  |  |
| 3500-5999 | 366 (24.3) | 631 (25.1) |  |  |
| 6000-9000 | 100 (6.6) | 227 (9.0) |  |  |
| >9000 | 113 (7.5) | 169 (6.7) |  |  |
| Occupation |  |  | 65.719 | **<0.001** |
| Government officer /teacher/healthcare provider | 20 (1.3) | 60 (2.4) |  |  |
| Factory/business/agriculture/service industry employee | 695 (46.1) | 882 (35.1) |  |  |
| Retired | 578 (38.4) | 1260 (50.2) |  |  |
| Other | 214 (14.2) | 309 (12.3) |  |  |
| Marital status |  |  | 22.459 | **<0.001** |
| Married/cohabitation | 1114 (73.9) | 2017 (80.3) |  |  |
| Single/widowed/divorced/separated | 393 (26.1) | 494 (19.7) |  |  |
| Current smoking |  |  | 9.234 | **0.002** |
| Yes | 280 (18.6) | 568 (22.6) |  |  |
| No | 1227 (81.4) | 1943 (77.4) |  |  |
| Current alcohol drinker |  |  | 2.002 | 0.158 |
| Yes | 114 (7.6) | 222 (8.8) |  |  |
| No | 1393 (92.4) | 2289 (91.2) |  |  |
| Tea-drinking habits |  |  | 5.894 | **0.016** |
| Yes | 708 (47.0) | 1279 (50.9) |  |  |
| No | 799 (53.0) | 1232 (49.1) |  |  |
| Exercise frequency |  |  | 29.314 | **<0.001** |
| Hardly ever | 362 (24.0) | 429 (17.1) |  |  |
| 1-3 times/month | 105 (7.0) | 194 (7.7) |  |  |
| 1-2 times/week | 126 (8.4) | 210 (8.4) |  |  |
| 3-5 times/week | 138 (9.2) | 254 (10.1) |  |  |
| Almost everyday | 776 (51.5) | 1424 (56.7) |  |  |
| Number of chronic diseases |  |  | 2.074 | 0.355 |
| 0 | 504 (33.4) | 885 (35.2) |  |  |
| 1 | 519 (34.4) | 869 (34.6) |  |  |
| ≥2 | 484 (32.1) | 757 (30.1) |  |  |
|  | **Mean (SD)** | **Mean (SD)** | *t* | *P* |
| Age (years) | 72.59 (2.67) | 71.24 (5.51) | -6.862 | **<0.001** |
| Depression | 0.99 (2.62) | 0.82 (2.62) | -1.893 | 0.061 |
| Anxiety | 1.03 (2.43) | 0.90 (2.18) | -1.735 | 0.083 |
| Insomnia | 3.37 (4.24) | 2.97 (3.81) | -2.996 | **0.003** |

Findings significant at the *P*<0.05 level are shown in bold. Chi-square tests and *t*-tests were used in the statistical analysis.
